# ANP32 proteins from ticks and vertebrates are key host factors for replication of Bourbon virus across species

**DOI:** 10.1128/jvi.00522-25

**Published:** 2025-05-14

**Authors:** Zhenyu Zhang, Ishmael D. Aziati, Thomas Nipper, Adrianus C. M. Boon, Andrew Mehle

**Affiliations:** 1 Medical Microbiology and Immunology, University of Wisconsin-Madison 5228, Madison, Wisconsin, USA; 2 Department of Medicine, Washington University School of Medicine in St. Louis 12275, St. Louis, Missouri, USA; 3 Department of Molecular Microbiology, Washington University School of Medicine in St. Louis 12275, St. Louis, Missouri, USA; 4 Department of Pathology and Immunology, Washington University School of Medicine in St. Louis 12275, St. Louis, Missouri, USA; Loyola University Chicago - Health Sciences Campus, Maywood, Illinois, USA

**Keywords:** orthomyxovirus, anp32, thogotovirus, Bourbon virus, virus:host interactions, influenza virus, tick, host range

## Abstract

Bourbon virus (BRBV) is a tick-borne virus in the genus *Thogotovirus* in the *Orthomyxoviridae* family. BRBV was initially identified as the presumptive causative agent of a fatal human infection in 2014 and has since been identified in ticks in the Midwest, Northeast, and Southern United States, with occasional spillovers into humans. However, little is known about how virus-host interactions impact their large host range. Here, we show that BRBV polymerase activity in human cells is completely dependent on cellular ANP32 proteins. BRBV polymerase activity was completely lost in cells lacking ANP32A and ANP32B, resulting in failed infections. BRBV polymerase activity was restored in the presence of ANP32 proteins from diverse hosts. Dhori virus and Thogoto virus, other related *Thogotovirus* members, retained high activity in the absence of ANP32 proteins, showing reduced dependence on these host factors. Interaction studies revealed that the BRBV polymerase trimer binds human ANP32A or ANP32B. Genetic analysis revealed that tick vectors for BRBV encode a single *ANP32* locus corresponding to *ANP32A*. Tick *ANP32A* produces multiple protein variants through alternative splicing and start-site selection, all of which enhance polymerase activity for *Thogotoviruses*. Unexpectedly, the BRBV polymerase was highly sensitive to changes at the N-terminus of ANP32, while it was insensitive to changes in the body of ANP32 that restrict the activity of influenza virus polymerases. Thus, ANP32A is a deeply conserved pro-viral cofactor, and *Thogotoviruses* show remarkable plasticity utilizing ANP32 homologs from different hosts separated by almost 1 billion years of evolution.

**IMPORTANCE:**

Viral polymerases rely on cellular cofactors to support efficient transcription of viral genes and replication of the viral genome. The RNA-dependent RNA polymerase of influenza virus, an orthomyxovirus, requires the cellular ANP32A or ANP32B proteins for genome replication. However, little is known about whether ANP32 proteins are required by other orthomyxovirus family members, like the tick-borne thogotoviruses. We show that thogotoviruses use ANP32 proteins from diverse hosts to enhance polymerase activity, including that encoded by the single *ANP32A* gene found in ticks. However, thogotovirus polymerase showed varying levels of dependence on ANP32 proteins, with some polymerases functioning at near full activity even in the absence of ANP32 proteins. Thus, ANP32 proteins are deeply conserved viral cofactors, with each virus displaying distinct patterns of ANP32 usage and requirements for function.

## INTRODUCTION

Bourbon virus (BRBV) is an emerging tick-borne RNA virus that was first detected in 2014 in a severely ill patient from Bourbon County, Kansas. This individual died 2 days later from complications of renal failure, acute respiratory distress syndrome, and cardiac arrhythmia (1). Serosurveillance of residents in Missouri and North Carolina identified BRBV-specific serum-neutralizing antibodies in 0.7% and 0.8% of individuals, respectively, suggesting that spillover of BRBV from ticks to humans is a relatively common, but unappreciated event (2, 3). The main vector for BRBV is the lone star tick (*Amblyomma americanum*), which is widely distributed throughout the central, eastern, southeastern, and south central United States, with tick surveillance programs detecting BRBV in these regions (4, 5). Recently, BRBV has also been detected in Asian long-horned ticks (*Haemaphysalis longicornis*), which is an invasive tick species with a growing distribution in the United States (6). BRBV virus has an expanding geographic and host range that is only likely to get larger as tick species occupy new areas in response to changing climate conditions.

Wildlife surveillance studies have identified BRBV-neutralizing antibodies in a wide range of mammalian species, including white-tailed deer, raccoons, dogs, horses, bobcats, red foxes, and coyotes (7, 8). There is no evidence of BRBV infection in opossums, and BRBV-specific antibodies have not been detected in avian species, suggesting some restrictions on BRBV’s host range. Yet, the potential barriers to cross-species transmission and host factors that control this process are not well defined.

BRBV is a negative sense-segmented RNA virus in the genus *Thogotovirus* in the family of *Orthomyxoviridae*. This genus includes other tick-borne viruses such as Thogoto virus (THOV), Dhori virus (DHOV), and Oz virus. Like influenza viruses, BRBV and related *Thogotoviruses* use a virally encoded RNA-dependent RNA polymerase for genome replication and transcription (9). The viral polymerase is a heterotrimer of the proteins PB2, PB1, and PA. The polymerase assembles with the minus-sense viral genome (vRNA) and nucleoprotein (NP) to form ribonucleoprotein complexes (RNPs) that scaffold enzymatic activity. Transcription occurs via a cap-snatching process whereby cellular 5′ 7 mG caps are appended to viral mRNAs. BRBV and THOV cap-snatching appears to acquire only the 7 mG cap and possibly one additional host residue, although the exact process is unclear (10, 11). This differs from influenza virus cap-snatching, which uses conserved cap-binding and exonuclease domains in the viral polymerase to obtain the 5′ 7 mG cap and ~13 nt of host mRNA that prime synthesis of viral messages (12). Structures and biochemistry suggest that these domains are non-functional for THOV, implicating a different cap-snatching process for *Thogotoviruses* (13, 14). Replication initiates in a primer-independent fashion to create full-length plus-sense cRNA intermediates that are then copied back to the minus-sense vRNA. While the viral polymerase contains all the enzymatic activities necessary for these transcription and replication, it is dependent on cellular cofactors to perform these activities during infection (15). Proteins in the ANP32 family were recently identified as essential cofactors for orthomyxoviruses like the influenza virus, where they assist genome replication (16–19). Cells lacking functional ANP32 proteins cannot support viral polymerase activity or infection by influenza viruses (20, 21). Furthermore, species-specific differences in ANP32A and ANP32B sequence or splicing alter their ability to be used as cofactors, restricting viral host range and driving adaptations in the viral polymerase (22–25).

BRBV is an emerging threat to human and animal health. The cellular cofactors that support or restrict cross-species transmission are poorly understood. Here, we show that ANP32 proteins are essential cofactors for BRBV polymerase activity and viral replication. This contrasts with other family members, THOV and DHOV, which show reduced dependence on ANP32 proteins. BRBV polymerase interacts with human ANP32A or ANP32B and is uniquely sensitive to changes at the N-terminus of these cofactors. We further identified the single ancestral locus of ANP32A in ticks and showed that multiple protein isoforms expressed from this gene support BRBV polymerase activity despite encoding variations that prevent its use by influenza virus polymerase. These data suggest that the BRBV polymerase engages ANP32 cofactors in a manner that is distinct from influenza virus polymerases. Moreover, our results show that Thogotoviruses promiscuously use ANP32 proteins from multiple species, likely contributing to their broad host range. Thus, ANP32 usage is a deeply conserved feature of *Orthomyxoviridae*.

## RESULTS

### BRBV polymerase activity and viral replication require either human ANP32A or ANP32B

**Fig 1:**
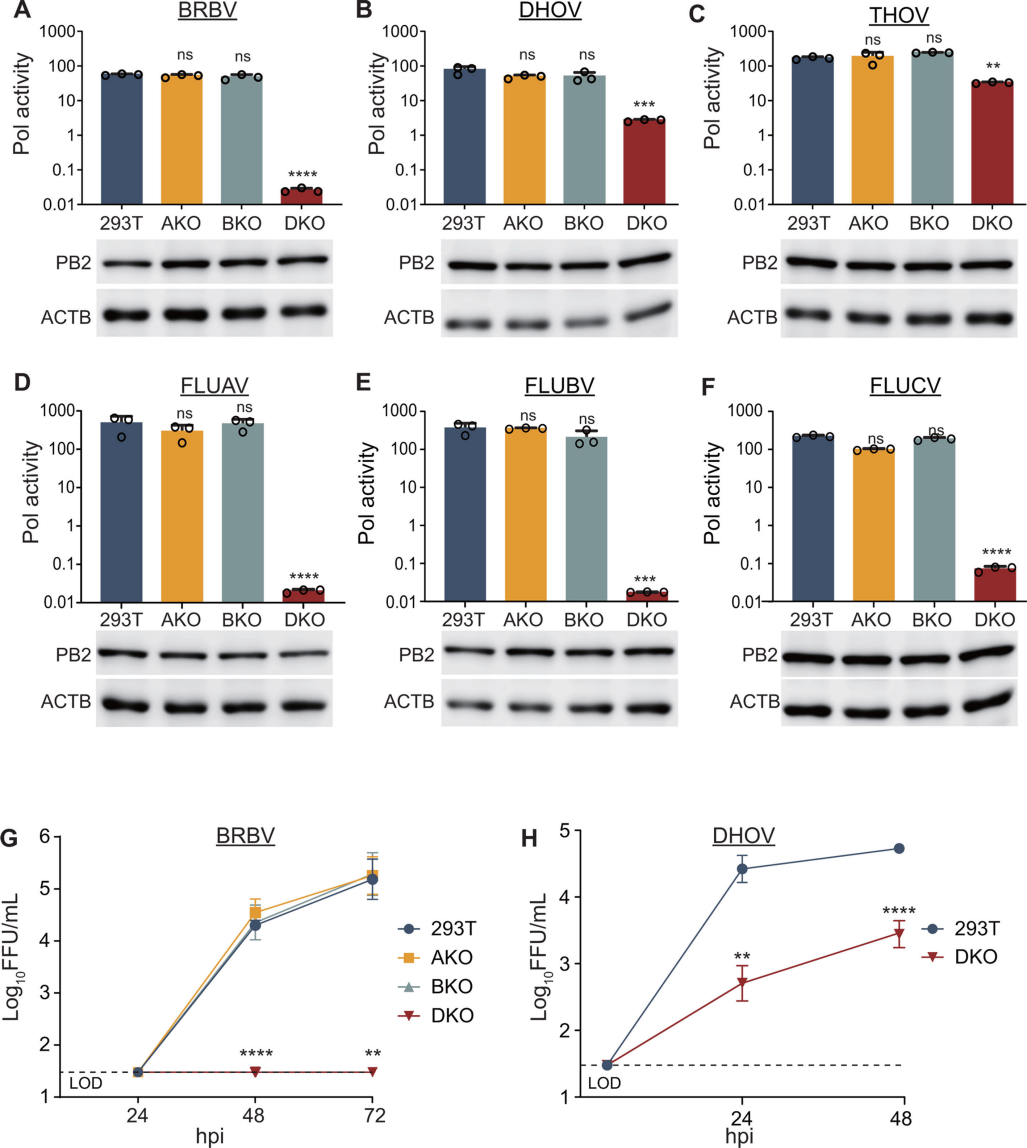
Human ANP32A and ANP32B are required host factors for thogotovirus polymerase activity and replication. (**A–F**) ANP32A and ANP32B increase polymerase activity for diverse orthomyxoviruses. Polymerase activity assays were performed in WT or knockout cells by expressing the polymerase proteins, nucleoprotein, and a viral firefly luciferase reporter for the indicated virus. Polymerase activity was normalized to an internal Renilla luciferase control and reported as the ratio of firefly/Renilla luciferase. FLUCV, influenza C virus. The polymerase subunit PB2 was expressed with a FLAG epitope tag. PB2 and the loading control β-actin were detected by western blotting. (**G and H**) Multicycle replication of BRBV (multiplicity of infection [MOI] = 0.1) or DHOV (MOI = 1) in 293T and knockout cells. Titers determined by focus-forming assay. LOD, limit of detection. (**A–F**) Data are mean of *n* = 3 ± sd and significance was assessed by a one-way analysis of variance (ANOVA) with a post hoc Dunnett’s multiple comparisons test against WT 293T cells. (**G and H**) Data are mean of *n* = 6 ± sd with comparisons to DKO cells made by a two-way ANOVA with a Tukey’s post hoc test. **P ≤ 0.01; ***P ≤ 0.001; ****P ≤ 0.0001; ns, not significant.

ANP32A and ANP32B are essential host factors for the viral polymerases from influenza A virus (FLUAV) and influenza B virus (FLUBV) in human cells (21, 24). Species-specific differences in ANP32A and ANP32B coding and splicing define influenza virus host range (18, 21–26). The role of these proteins during replication and spillover of BRBV, a distantly related orthomyxovirus in the genus *Thogotovirus,* is unknown. We therefore investigated BRBV polymerase activity in human cells, using WT cells or engineered *ANP32A*-knockout cells (AKO), *ANP32B*-knockout cells (BKO), and dual knockout cells (DKO). Polymerase activity assays were performed by expressing the viral polymerase trimer PB1, PB2, and PA, the viral NP, and a vRNA-like reporter encoding firefly luciferase. In the presence of functional polymerases and NP, viral RNPs assemble to replicate and transcribe the reporter. Reporter activity then serves as a proxy for the composite output of polymerase activities. BRBV polymerase is active in human cells (Fig. 1A), consistent with its ability to infect and cause disease in mammals (7, 27). BRBV polymerase activity is unchanged in AKO and BKO cells, but is reduced to background levels in DKO cells (Fig. 1A). PB2 levels were equivalent in all cells, indicating changes in ANP32 expression did not alter viral protein expression. Thus, either human ANP32A (huANP32A) or ANP32B (huANP32B) is necessary for BRBV polymerase activity. Our cells retain the *ANP32E* gene, yet the effectively complete loss of polymerase activity suggests that BRBV polymerase is unable to use human ANP32E (huANP32E).

To establish the generalizability of our findings, we extended these experiments to polymerases from other members of the *Thogotovirus* genus. Similar to BRBV, DHOV and THOV polymerases were highly active in AKO and BKO cells with output indistinguishable from that in WT cells (Fig. 1B and C). However, they retained significant levels of activity in DKO cells exhibiting reduced dependence on huANP32A and huANP32B proteins; whereas BRBV activity decreased over 1,000-fold in DKO cells, DHOV was reduced by only 10-fold, and THOV decreased by approximately fivefold. Confirming prior reports, FLUAV and FLUBV polymerase required either ANP32A or ANP32B (Fig. 1D and E) (21, 24). Influenza C virus (FLUCV) also required either ANP32A or ANP32B for high activity (Fig. 1F and [17]). FLUCV appeared to have low levels of residual activity in DKO cells, but whether this is analogous to the phenotypes exhibited by THOV and DHOV is unclear. In all cases, the viral polymerase subunit PB2 was expressed at equivalent levels in the presence or absence of ANP32 proteins.

Polymerase activity is absolutely required for viral replication. We therefore tested the importance of ANP32 proteins during BRBV infection. Multicycle replication assays showed that BRBV replicates to high levels in WT, AKO, and BKO cells with titers nearly identical at all time points (Fig. 1G). However, viral replication was not observed above the level of detection in DKO cells. DHOV replicated in WT cells and showed significant levels of replication in DKO cells (Fig. 1H), paralleling the polymerase activity assays. Thus, either huANP32A or huANP32B is necessary for BRBV polymerase activity and successful infections, whereas DHOV and THOV show reduced dependence.

**Fig 2:**
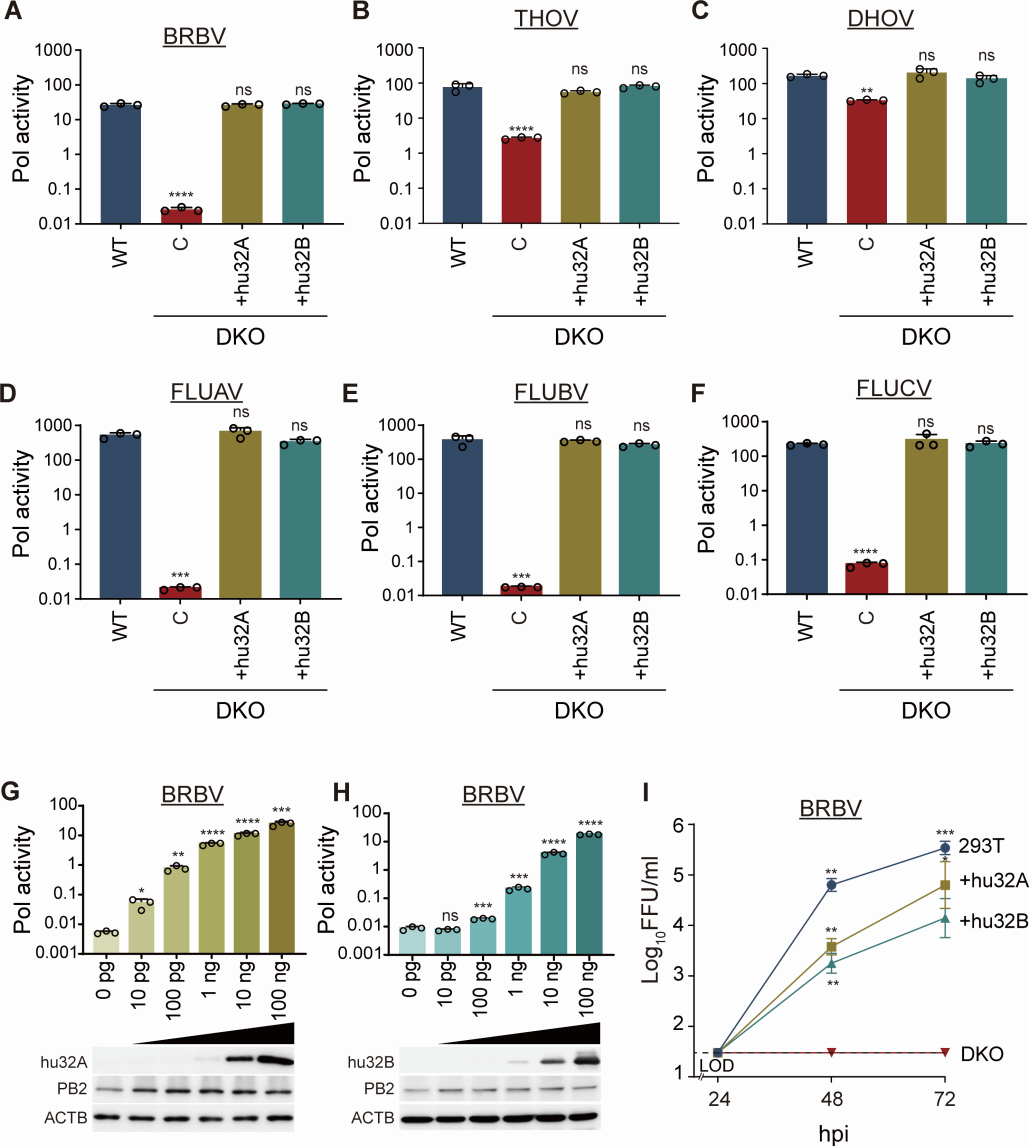
Human ANP32A or ANP32B is sufficient for thogotovirus polymerase activity and replication. (**A–F**) Polymerase activity was measured in WT 293T cells, DKO cells, or DKO cells stably complemented with human ANP32A or ANP32B. Polymerase activity assays were performed by expressing the polymerase proteins, nucleoprotein, and a viral firefly luciferase reporter for the indicated virus. Polymerase activity was normalized to an internal *Renilla* luciferase control. (**G and H**) Human ANP32A and ANP32B enhance BRBV polymerase activity in a dose-dependent manner. Polymerase activity was measured in DKO cells as in (**A**) in the presence of increasing amounts of expression vector for (**G**) human ANP32A or (**H**) human ANP32B. PB2-FLAG, ANP32-FLAG, and the loading control β-actin were detected by western blotting. (**I**) Multicycle replication of BRBV in 293T cells, DKO cells, or DKO cells stably complemented with human ANP32A or human ANP32B (MOI = 0.01). Titers were determined by focus-forming assay. (**A–H**) Data are mean of *n* = 3 ± sd. Significance was assessed by a one-way ANOVA with a post hoc Dunnett’s multiple comparisons test against WT 293T cells (**A–F**) or empty vector controls (**G and H**). (**I**) Data are mean of *n* = 6 ± sd with comparisons to DKO cells made by a two-way ANOVA with a Tukey’s post hoc test. *P ≤ 0.05; **P ≤ 0.01; ***P ≤ 0.001; ****P ≤ 0.0001; ns, not significant.

We next tested the sufficiency of ANP32 proteins. DKO cells were complemented by stable expression of huANP32A or huANP32B and used for polymerase activity assays and infections. Expression of either huANP32A or huANP32B increased BRBV polymerase activity >1,000-fold, fully restoring it to levels present in parental WT cells (Fig. 2A). This suggests that the DKO phenotype is due to the loss of ANP32A and ANP32B and not background effects. Similar results were detected for THOV, DHOV, FLUAV, FLUBV, and FLUCV (Fig. 2B through F). The effects of complementation were muted for THOV and DHOV because, as before, they exhibited more modest defects in DKO cells. huANP32A or huANP32B supported BRBV polymerase activity in a dose-dependent manner (Fig. 2G and H). Moreover, complementation of DKO with huANP32A or huANP32B rescued viral replication (Fig. 2I). Together, these data show that huANP32A or huANP32B is sufficient for BRBV polymerase activity and viral replication.

### Functional interactions between ANP32 and BRBV polymerase

**Fig 3:**
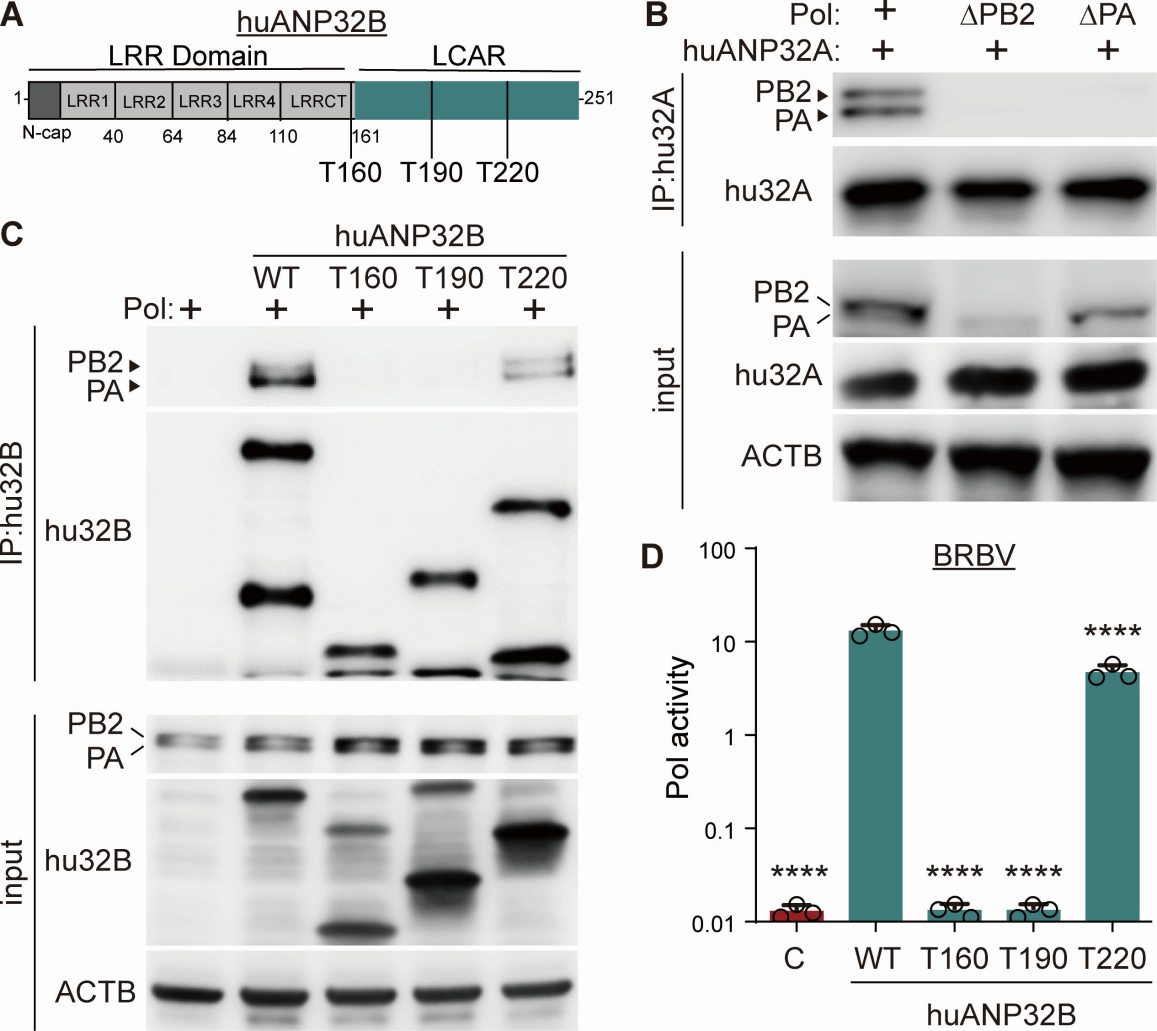
BRBV polymerase binds ANP32 and requires its C-terminal low complexity acidic region. (**A**) Schematic diagram of full-length human ANP32B and three C-terminal truncations at amino acid 160 (T160), 190 (T190), and 220 (T220). Domains and boundaries are indicated. LRR = leucinerich repeat; LCAR = low complexity acidic region. T160, T190, and T220 = terminal amino acids in C-terminal truncations. (**B**) Human ANP32A (hu32A) interacts with the BRBV polymerase trimer. Human ANP32A-FLAG was expressed in cells with the BRBV polymerase or versions lacking PB2 or PA. Human ANP32A-FLAG was immunoprecipitated from whole-cell lysate and probed for huANP32A or V5-tagged PB2 and PA. Input samples were blotted as an expression control. β-actin served as a loading control. (**C**) The LCAR of human ANP32B (hu32B) is important for interaction with the BRBV polymerase. Polymerase proteins were expressed in DKO cells in the presence of human ANP32B-FLAG, the indicated truncations, or a negative control. Interaction with the polymerase was tested by immunoprecipitating huANP32B-FLAG from whole-cell lysate and probing for huANP32B and co-precipitating PB2 and PA that contained a C-terminal V5 tag. Input samples were blotted as an expression control. β-actin served as a loading control. (**D**) BRBV polymerase activity assays were performed in DKO cells expressing WT or truncated huANP32B, or an empty vector control. Polymerase activity was normalized to an internal Renilla luciferase control. Data are mean of *n* = 3 ± sd. Significance was assessed by a one-way ANOVA with a post hoc Dunnett’s multiple comparisons test against cells expressing WT huANP32B. ****P ≤ 0.0001.

ANP32 proteins contain an N-terminal leucine-rich region (LRR) and a C-terminal low-complexity acidic region (LCAR) (Fig. 3A). The LRR of ANP32 proteins forms a compact domain that bridges influenza virus polymerase complexes to form higher-order structures that mediate genome replication, while the LCAR has a less defined structure spanning the polymerase dimers (16, 17, 19). Co-immunoprecipitations revealed that the BRBV polymerase complex interacts with huANP32A (Fig. 3B). This interaction required the intact BRBV polymerase trimer, as huANP32A failed to co-precipitate when the PB2 or PA subunits were missing. We used a series of truncations to identify the functional domains required for activity and interactions with the BRBV polymerase. Like huANP32A, huANP32B specifically co-precipitated with the BRBV polymerase (Fig. 3C). A truncation at residue 160 (T160) that removed the LCAR disrupted interaction with BRBV polymerase. A truncation at residue 190 (T190) that retained ~1/3 of the LCAR also failed to bind the BRBV polymerase. The T220 truncation that included ~2/3 of the LCAR bound the BRBV polymerase, although not as well as full-length huANP32B. Results from interaction studies correlated well with polymerase activity assays. huANP32B T160 and T190 did not support BRBV polymerase activity, whereas T220 showed high levels of activity just below that conferred by full-length huANP32B (Fig. 3D). While structures show that the LRR makes many of the shared contacts between viral polymerase and ANP32, our assays show that the LCAR is critical for stable interactions and activity for the BRBV polymerase.

### Ticks encode a single ancestral ANP32 locus that supports BRBV polymerase activity

**Fig 4:**
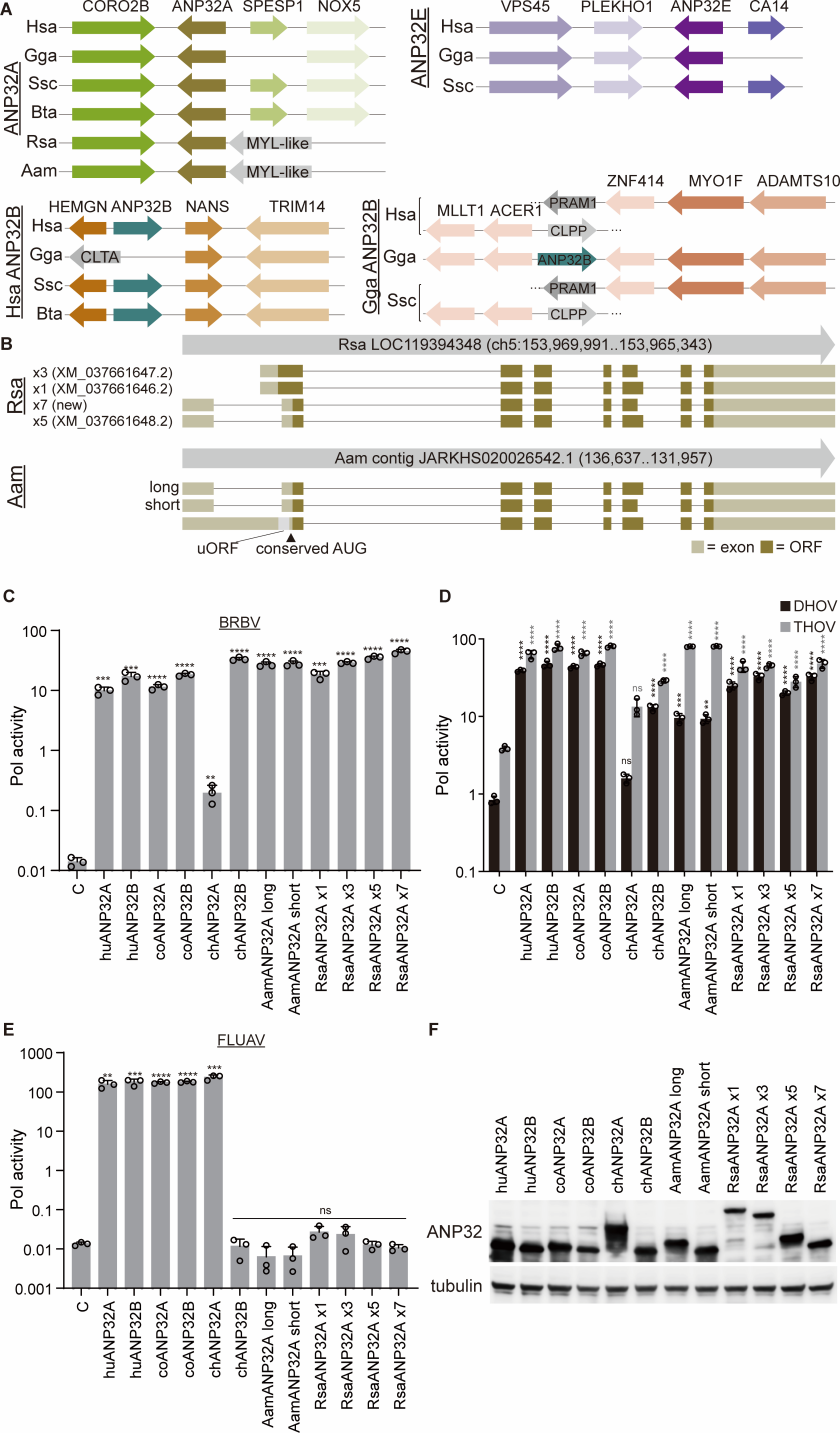
Ticks encode a single ancestral locus expressing ANP32A variants that support BRBV polymerase activity. (**A**) Syntenic gene blocks were identified for ANP32 genes, revealing a single ancestral locus present in the lone star (Aam) ticks and brown dog (Rsa) ticks. Comparisons were made to humans (Hsa, *Homo sapiens*), chickens (Gga), swine (Ssc), and cows (Bta). (**B**) *De novo* transcriptome assembly identifies multiple splice variants of tick ANP32. The genomic locus for Rsa and Aam ticks is indicated, as well as accession numbers for previously cataloged transcript variants. Exons are shown as boxes with non-coding regions in pink, open reading frames in red, and a small upstream open reading frame (uORF) in gray. The conserved start site present in vertebrate ANP32A is indicated. (**C**) BRBV polymerase activity assays were performed in DKO cells expressing the viral polymerase, nucleoprotein, and a viral reporter as well as the indicated ANP32A or ANP32B from humans (hu), cows (co), chickens (ch), lone star ticks (Aam), or brown dog ticks (Rsa). Naming of tick variants corresponds to those diagrammed in (B). Activity was normalized to an internal Renilla luciferase control. (**D**) Polymerase activity assays were performed for DHOV and THOV as in (C). (**E**) Activity of the FLUAV polymerase in cells expressing diverse ANP32 proteins was measured as described for (C). For (C)–(E), data are mean of *n* = 3 ± sd. Significance was assessed by a one-way ANOVA with a post hoc Dunnett’s multiple comparisons test against the empty vector. **P ≤ 0.01; ***P ≤ 0.001; ****P ≤ 0.0001; ns, not significant. (**F**) Expression of ANP32 proteins was detected by western blot. Tubulin was probed as a loading control.

While the importance of ANP32 family members has been investigated in detail for influenza viruses, little is known about their expression in ticks or use by tick-borne *Thogotoviruses*. Humans express three ANP32 genes (*ANP32A*, *ANP32B,* and *ANP32E*) (28). Our genetic analysis of *Amblyomma americanum* (*Aam*), the tick vector for BRBV, and *Rhipicephalus sanguineus* (*Rsa*)*,* a known vector for other thogotoviruses, revealed that ticks encode only a single *ANP32* gene (Fig. 4A). Synteny indicates that this is the locus that became *ANP32A* in humans and other orthomyxovirus hosts like chickens (*Gallus gallus* [Gga]), pigs (*Sus scrofa* [Ssc]), and cows (*Bos taurus* [Bta]). Analogous syntenic blocks for *ANP32B* and -*E* were not found in the tick genome.

We performed *de novo* transcriptome assembly to identify the actual transcripts and proteins expressed from the tick *ANP32A* locus (Fig. 4B). Multiple splice variants were identified in both tick species, revealing that differential splicing is a deeply conserved feature of the *ANP32A* gene (22, 25).

*Rsa* and *Aam ANP32A* express proteins that initiate near the conserved start site (Fig. 4B). Interestingly, *Rsa* ticks also express variants with highly unusual N-terminal extensions not common in vertebrates (Fig. 4B). One of the *Aam* transcripts also encoded an AUG 5′ to the conserved start site, but in this case, it created a small upstream ORF (uORF) as opposed to a N-terminally extended version of the protein (Fig. 4B). In *Rsa ANP32A*, differential splicing in transcript x3 and x7 removed sequences that code for residues toward the C-terminus of the LCAR. A similar deletion was observed amongst our newly defined *Aam ANP32A* transcripts. This indel is distinct from the duplication of an exon in chicken ANP32A that confers its species-specific activity (18). A recent report claimed to identify and test the activity of *Rsa* “ANP32A” or “ANP32B” (XP_037517575.1 and XP_037517574.1, respectively) (13), but close inspection of these proteins revealed that they are not encoded by distinct genes. Rather, they are the splice variants x3 and x1 that map to the single *ANP32A* locus in the *Rsa* genome (Fig. 4B). We find that automated annotation of the *Rsa* tick and other tick genomes appears to have resulted in the misidentification of *ANP32* protein genes that are annotated as distinct *ANP32* family members across many tick species. In reality, they are spliceoforms that all map to the same ancestral *ANP32A* locus. These results identified canonical and non-canonical tick ANP32, all encoded by a single locus where splicing and start-site usage diversify the proteins that are produced.

To test the role of *bona fide* tick ANP32A as a viral cofactor, we performed polymerase activity assays using our DKO cells as a “clean slate” to measure the activity of heterologous ANP32 proteins. Cells were complemented with ANP32 proteins from humans, chickens, cows, or ticks. BRBV polymerase was highly active in cells expressing ANP32A or ANP32B from humans and cows (Fig. 4C). Chicken ANP32B also conferred high polymerase activity, whereas the enhancement by chicken ANP32A was rather modest, only ~10-fold over background. All the tick ANP32A variants from both *Aam* and *Rsa* ticks supported high levels of BRBV polymerase activity (Fig. 4C). The species of origin, the presence of an N-terminal extension, or differential splicing in the LCAR of tick isoforms did not cause drastic changes in BRBV polymerase activity. Similar trends were detected in polymerase activity assays for DHOV and THOV, although, as before, these polymerases were less dependent on ANP32A than BRBV (Fig. 4D). In addition, the DHOV polymerase was poorly supported by *Aam* ANP32A proteins. Like BRBV, FLUAV polymerase utilized human and cow ANP32A and ANP32B (Fig. 4E). In contrast, and paralleling prior results, FLUAV polymerase was supported by chicken ANP32A, but not chicken ANP32B (23, 24). Furthermore, none of the tick ANP32A proteins were functional for FLUAV polymerase (Fig. 4E). This was not caused by changes in expression, as all ANP32 proteins were expressed at roughly similar levels (Fig. 4F).

### BRBV polymerase has distinct requirements of ANP32A

**Fig 5:**
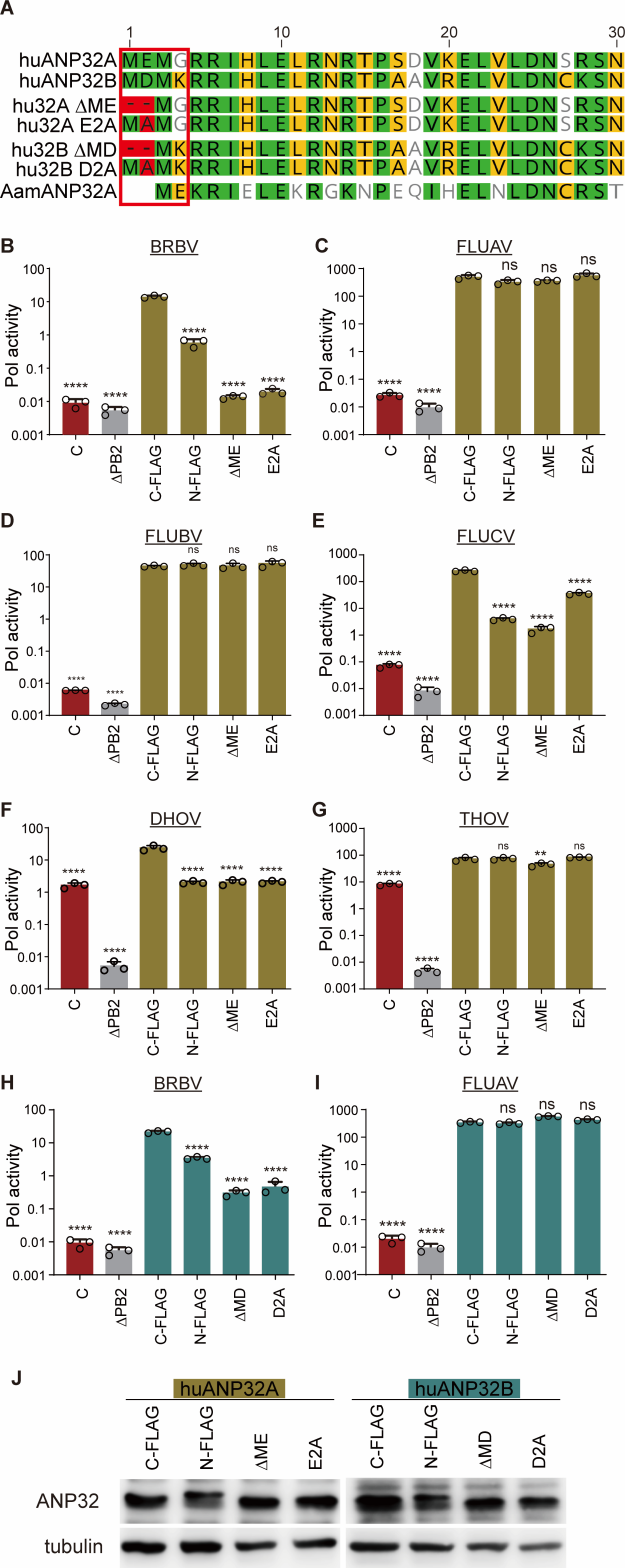
BRBV polymerase activity is sensitive to changes at the N-terminus of ANP32. (**A**) Alignment of the N-terminus of human ANP32A (hu32A), ANP32B (hu32B), and lone star tick (Aam) ANP32A (Aam 32). Mutants used below are highlighted in red. (**B–D**) Changes to the N-terminus of ANP32 disrupt its ability to support BRBV polymerase. (**B–G**) Polymerase activity assays for the indicated polymerase were performed in DKO cells expressing huANP32A with a C-terminal FLAG tag (C-FLAG), an N-terminal FLAG tag (N-FLAG), a C-terminal FLAG tag with an N-terminal deletion (∆ME) or mutation (E2A), or an empty vector control. Polymerase lacking the PB2 subunit (∆PB2) served as a negative control. (**H–I**) Polymerase activity assays were performed with the indicated polymerase in DKO cells expressing huANP32B with a C-terminal FLAG tag (C-FLAG), an N-terminal FLAG tag (N-FLAG), a C-terminal FLAG tag with an N-terminal deletion (∆MD) or mutation (D2A), or an empty vector control. Polymerase lacking the PB2 subunit (∆PB2) served as a negative control. For all, polymerase activity was normalized to an internal Renilla luciferase control. Data are mean of *n* = 3 ± sd. Significance was assessed by a one-way ANOVA with a post hoc Dunnett’s multiple comparisons test against huANP32 C-FLAG. ****P ≤ 0.0001; ns, not significant. (**J**) Representative blot of ANP32 proteins and variants confirming equivalent expression. Tubulin was probed as a loading control.

Canonical ANP32A isoforms in ticks (i.e., *Rsa* x1 and x3 and *Aam* short) initiate near the conserved start site but use a different AUG, two codons downstream from that shared by humans, swine, chickens, and cows (Fig. 5A). Regardless of start site, all canonical isoforms initiate with a methionine followed by an acidic residue. We therefore investigated whether changes at the N-terminus of ANP32 affect its function as a viral cofactor. Polymerase activity assays were performed in DKO cells expressing huANP32A with changes at the N-terminus. Appending a FLAG epitope tag to the N-terminus of huANP32A reduced BRBV polymerase activity ~10-fold compared to protein with a C-terminal tag (Fig. 5B). In contrast to BRBV, N- and C-terminally tagged huANP32A supported FLUAV polymerase at similar levels (Fig. 5C). Epitope tags add artificial sequence to the protein, thus we focused on natural variation between humans and ticks at the N-terminus of ANP32A (Fig. 5A). We expressed a human ANP32A variant that initiated at the same site as tick ANP32A, removing the first two amino acids of the human protein (∆ME). We also mutated human ANP32A to remove the conserved acidic residue at the second position (E2A). Human ANP32A ∆ME and E2A completely failed to support the BRBV polymerase, with activity reduced to background levels (Fig. 5B), while these changes had no effect on FLUAV polymerase, which retained high activity (Fig. 5C). FLUBV and THOV polymerases were also unaffected by changes at the N-terminus of huANP32A, while FLUCV and DHOV polymerases were sensitive, showing reduced activity when the N-terminus was modified or deleted (Fig. 5D through G). We repeated these experiments using human ANP32B and the analogous mutants ∆MD and D2A (Fig. 5H and I). We obtained similar results where BRBV polymerase was sensitive to changes to the N-terminus of ANP32B, whereas FLUAV polymerase was unaffected. All of the ANP32 variants were expressed at equivalent levels (Fig. 5J).

**Fig 6:**
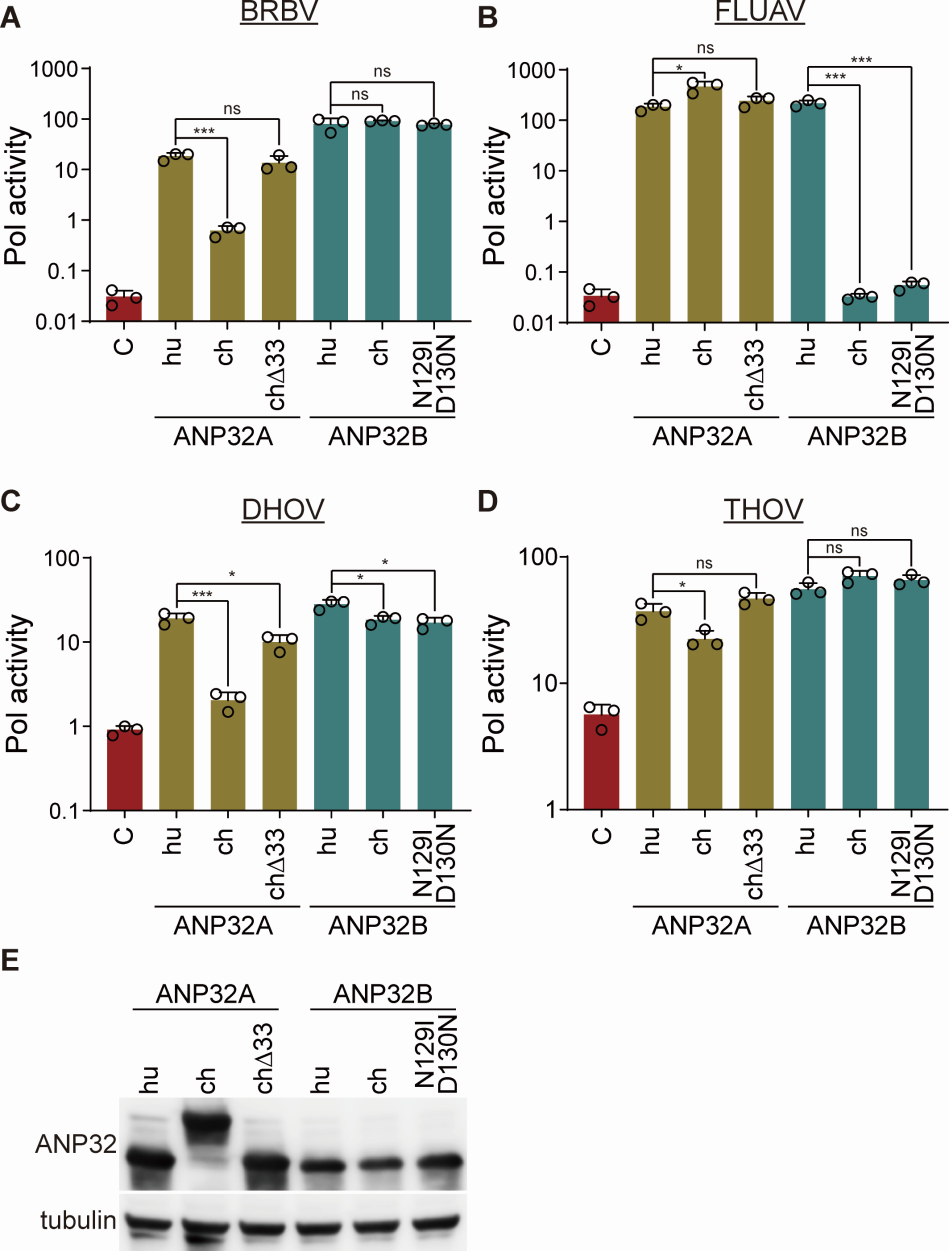
The impact of adaptive variants in chicken ANP32A and ANP32B differs between BRBV and FLUAV polymerases. (**A–D**) Polymerase activity assays were performed in DKO expressing the indicated viral polymerase, nucleoprotein, and a viral reporter. Cells were complemented with human (huANP32), chicken (chANP32), chANP32A lacking residues 175-207 (ch∆33), or mutant huANP32B N129I/D130N proteins. Activity was normalized to an internal Renilla luciferase control. Data are mean of *n* = 3 ± sd. Significance was assessed by a one-way ANOVA with a post hoc Dunnett’s multiple comparisons. ANP32A variants were compared against huANP32A, and ANP32B variants were compared against huANP32B. *P ≤ 0.05; ***P ≤ 0.001; ns, not significant. (**E**) Representative blot confirming expression of ANP32 variants. Tubulin was detected as a loading control.

Polymorphisms in ANP32 proteins impact their ability to be exploited by viral polymerases (29). For example, chANP32A is an essential cofactor, but chANP32B generally does not support influenza virus polymerase (Fig. 4E and [23, 24]). This difference in activity maps to polymorphisms at residues 129 and 130 in the last LRR domain (LRR4); chANP32A encodes N129 D130, whereas chANP32B encodes I129 N130. These changes alone are sufficient to alter activity (23, 24). Tick ANP32A encodes E124 N125 at the equivalent positions, providing an explanation for why tick ANP32As do not support FLUAV polymerase (Fig. 4E). We were surprised that BRBV polymerase functioned in the presence of chicken ANP32B but not ANP32A. The other major difference between the chicken proteins is a large 29-33 amino acid insertion in ANP32A caused by an exon duplication. This insertion is unique to birds and confers species-specific activity for FLUAV polymerases (18). To test the importance of these differences, we created a huANP32B protein with the chicken-style isoleucine at residue 129 and asparagine at residue 130 (huANP32B N129I D130N) and a chANP32A mutant that deletes the repeated region (chANP32A∆33). Polymerase activity assays were performed in cells expressing WT or mutant proteins. Chicken ANP32A provided minimal support for the BRBV polymerase, but this was dramatically enhanced by removal of the repeat in chANP32A∆33, which supported activity comparable to huANP32A (Fig. 6A). Human FLUAV polymerase was unaffected by changes to chANP32A (Fig. 6B), consistent with its ability to use ANP32A variants with or without the repeat (22). Mutations in ANP32B had no effect on BRBV polymerase activity, with human, chicken, and the human N129I D130N mutant all supporting high levels of activity (Fig. 6A). Conversely, human FLUAV polymerase was highly sensitive to the introduction of the avian-style residues, where polymerase activity was at background levels in cells expressing huANP32B N129I D130N (Fig. 6B). Similar patterns were detected in assays using the DHOV and THOV polymerase, where these proteins exhibited lower activity with chANP32A compared to promiscuous use of the other ANP32 proteins or mutants (Fig. 6C–D). All of the ANP32 variants expressed at levels comparable to the corresponding WT proteins (Fig. 6E).

**Fig 7:**
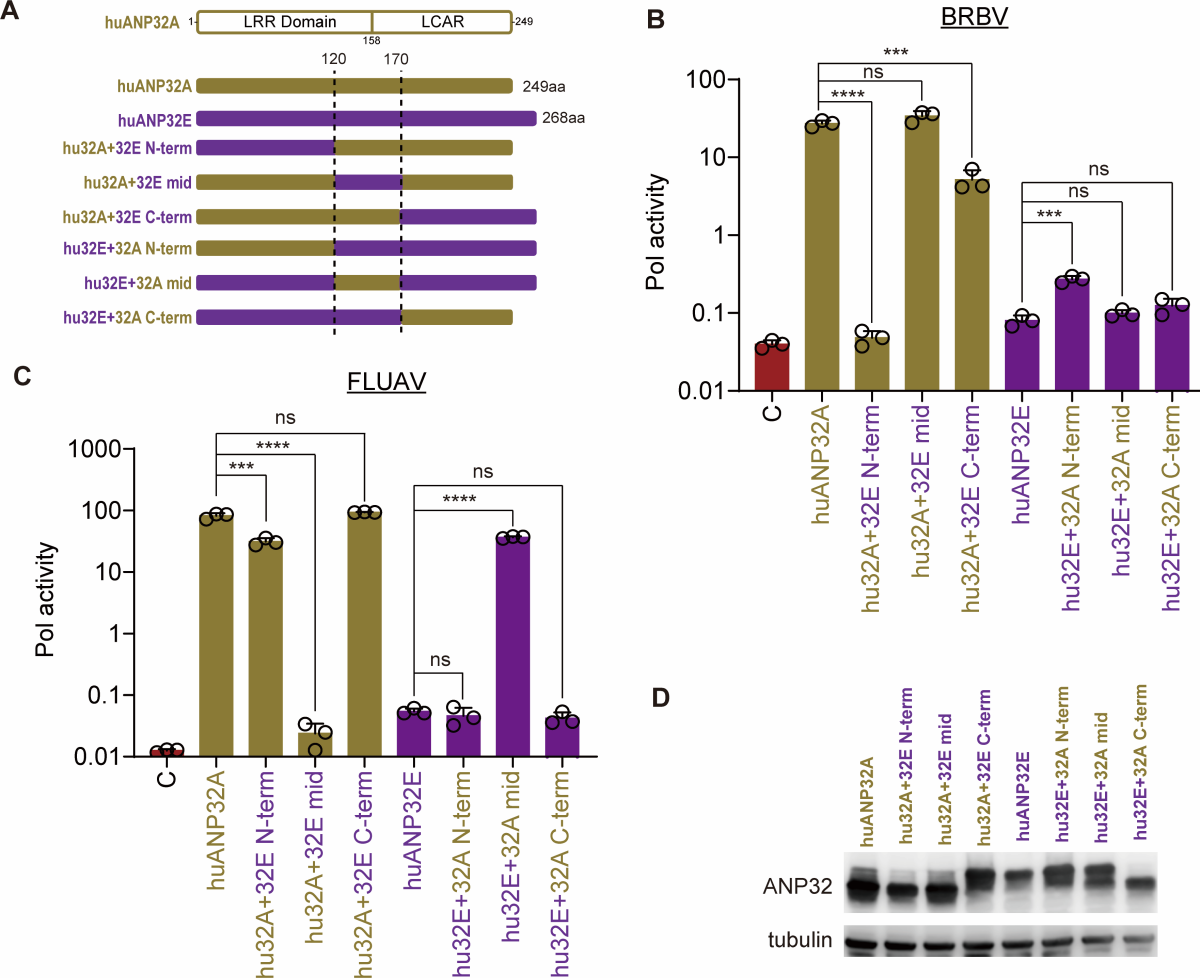
Amino acid variations in the N-terminus and central regions of ANP32E preclude its use by BRBV or FLUAV, respectively. (**A**) Diagram of chimeric proteins between human ANP32A and ANP32E. Fusions were made after residues 120 and 170. (**B**) BRBV polymerase, nucleoprotein, and a viral reporter were expressed in DKO cells with the indicated human ANP32A, ANP32E, or chimeric clone. Activity was normalized to an internal Renilla luciferase control. (**C**) FLUAV polymerase activity was measured as in (B). (**D**) Representative blot showing expression of ANP32 proteins and chimeras. Tubulin was detected as a loading control. Data are mean of *n* = 3 ± sd. Significance was assessed by a one-way ANOVA with a post hoc Dunnett’s multiple comparisons test against human ANP32A or ANP32E. ***P ≤ 0.001; ****P ≤ 0.0001; ns, not significant.

ANP32E proteins cannot normally be used as cofactors for FLUAV polymerases. However, huANP32E does provide limited support for FLUBV polymerase and for FLUAV polymerases with adaptations that arise during growth in DKO cells (20, 30). One of the determinants is the presence of a glutamate at residue 129 in huANP32E, which is normally asparagine in the huANP32A and huANP32B that support polymerase activity (20). Given that BRBV was insensitive to changes at residue 129 in ANP32B, we investigated whether BRBV can utilize huANP32E and mapped this function with a series of chimeras (Fig. 7A). Expression of huANP32E did not confer activity to the BRBV polymerase, with levels barely above background levels (Fig. 7B). Chimeras between human ANP32A and ANP32E revealed that the sequence at both ends of ANP32E confers this loss of function. Human ANP32A with the N-terminus derived from ANP32E completely lost the ability to support the BRBV polymerase, while a chimera with the C-terminus of ANP32E was ~10-fold less active than ANP32A. Conversely, adding the N-terminus of ANP32A to ANP32E caused a small increase, suggesting additional residues are needed to confer full activity. A different pattern was detected for FLUAV polymerase, where residues 120-170 determined if huANP32A supported polymerase activity (Fig. 7C). All of the ANP32 chimeras expressed at roughly equivalent levels (Fig. 7D). Together, these data show that BRBV and other *Thogotoviruses* have unique requirements for ANP32 activity—an unusual sensitivity to changes at the N-terminus but a general indifference to residues 129/130 that control function for influenza virus polymerases—that may affect pathogenicity and host range.

## DISCUSSION

The BRBV polymerase directs replication of the viral genome and transcription of viral messages. Here we show that ANP32 proteins from a diverse range of hosts, including ticks, are essential cellular cofactors for BRBV polymerase activity and subsequent viral replication. This contrasts with other *Thogotovirus* members, THOV and DHOV, whose polymerases were much less dependent on the presence of ANP32 proteins. *Thogotoviruses* are transmitted via tick vectors. Genetic analyses revealed that ticks encode a single ancestral *ANP32* locus corresponding to the gene that became *ANP32A* in vertebrates and is exploited by other orthomyxoviruses. This single gene produces multiple protein variants through alternative splicing and start-site selection, all of which enhance polymerase activity of *Thogotovirus* members. Functional analysis revealed that BRBV engages ANP32 proteins in a manner that is distinct from other orthomyxoviruses. Together, this work shows that ANP32 proteins are deeply conserved cellular cofactors, with each virus genus displaying different patterns of ANP32 usage and interactions.

Influenza viruses use ANP32 proteins during genome replication. The influenza virus polymerase forms an asymmetric dimer during genome replication. Dimerization is aided by ANP32A or ANP32B, which bridge the replicative polymerase with a polymerase that will encapsidate the newly made genomic RNA (17). The N-terminal LRR domain of ANP32A or ANP32B primarily contacts the encapsidating polymerase of FLUAV and FLUBV, while the binding site for the LRR domain of ANP32A spans both polymerases for FLUCV (16, 17, 31). The C-terminal LCAR domain takes a less constrained path that differs between FLUAV, FLUBV, and FLUCV following a basic groove made by both polymerases in the asymmetric dimer (16, 31). These interactions are key to directing genome replication. How BRBV polymerase functions and uses ANP32A or ANP32B is unclear, but parallels can be drawn from recent structures that provide a comprehensive overview of the biochemical mechanisms of the related THOV polymerase (13). Like the influenza virus, genome replication is performed by an asymmetric dimer of the THOV polymerase. Unlike the influenza virus, the THOV asymmetric dimer is less dependent on ANP32A or ANP32B, stably assembling without either protein, perhaps explaining the reduced dependence of THOV and DHOV on ANP32 proteins (Fig. 1B, C, 2B, and C). BRBV polymerase requires ANP32 proteins, implying that the BRBV asymmetric dimer interface is weaker than that for THOV and DHOV and uses ANP32 proteins to stabilize the replication platform. Thus, in this regard, the BRBV polymerase behaves more like influenza virus polymerases as it is completely dependent on ANP32A or ANP32B for activity (Fig. 1 and 2). BRBV polymerase most closely mirrors the FLUCV polymerase with similar patterns of activity in the presence of ANP32 mutants (Fig. 5).

The BRBV polymerase also differs from the influenza virus polymerase (Fig. 5 and 6). BRBV polymerase was insensitive to polymorphisms at residues 129 and 130 in ANP32A and ANP32B that impair FLUAV and FLUBV polymerase activity (23, 24). Conversely, chicken ANP32A only partially supported BRBV polymerase, while it is highly active for FLUAV. This lack of activity was mapped to an insertion present in chicken ANP32A that is critical for its ability to support the activity of chicken FLUAV polymerase (18). In addition, BRBV polymerase was uniquely sensitive to changes at the N-terminus of ANP32A and B. It is curious that small additions or deletions at the N-terminus of ANP32A or ANP32B impair BRBV polymerase activity, yet natural variants in ticks that use an upstream start codon to produce proteins with long N-terminal extensions all provided high levels of support for BRBV. This suggests that it is not the length of the N-terminus, *per se*, but perhaps other features such as amino acid sequence or N-terminal acetylation. BRBV polymerase interacts with ANP32 proteins in a manner that is distinct from other *Thogotoviruses* and influenza viruses.

**Fig 8:**
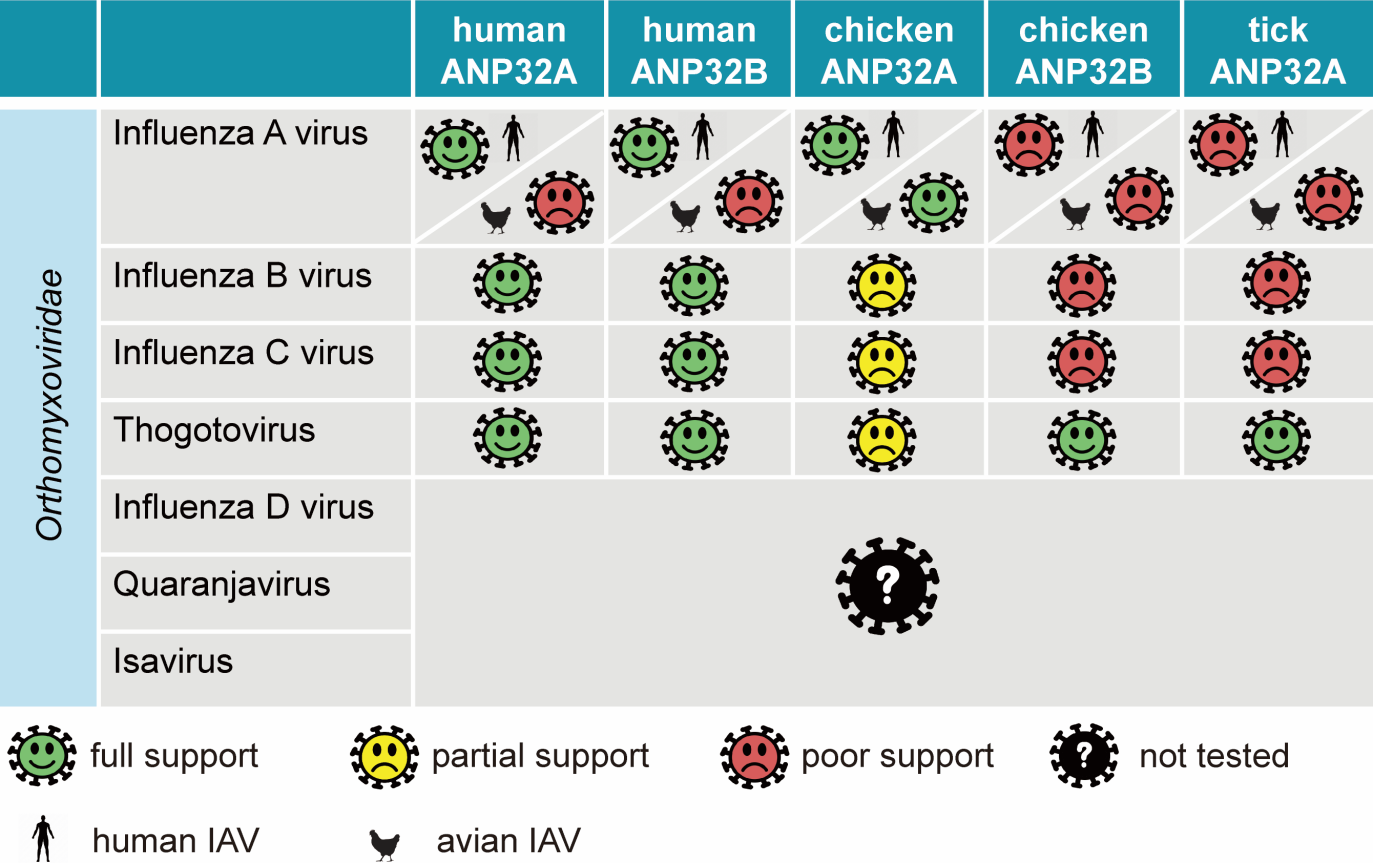
ANP32 usage is a deeply conserved feature of Orthomyxoviridae. Schematic of the relationship between different species of ANP32 proteins and *Orthomyxoviridae* family members, highlighting the promiscuous usage by *Thogotoviruses*.

*ANP32* coding variations and differential splicing establish viral host range for influenza viruses (15). Our data reveal that *Thogotoviruses* use ANP32 proteins encoded by diverse species (Fig. 8). This corresponds well to the incredibly broad host range of *Thogotoviruses*. BRBV virus replicates in and is transmitted by tick vectors (32). BRBV was originally isolated from an infected person, infects mice in experimental settings, and seroprevalence surveys indicate BRBV infects domesticated and wild mammals, including dogs, horses, raccoons, white-tailed deer, bobcats, red foxes, and coyotes (1, 7, 8, 27). This suggests that ANP32 proteins in these species are capable of supporting BRBV polymerase activity, many of which were confirmed in experiments here. By contrast, BRBV antibodies have not yet been detected in avian hosts. But our data show that BRBV can utilize chicken ANP32B, suggesting that ANP32 usage is not a limiting factor for replication in birds. Ticks that vector BRBV, the lone star tick and especially the Asian longhorned tick, will feed on birds (33–35); thus, the potential to transmit BRBV to avian hosts exists. Whether the failure to detect BRBV infections in birds reflects a true barrier to cross-species transmission or simply limitations of current surveillance data remains to be determined.

In summary, our data define the repertoire of tick ANP32A proteins that support polymerase activity, revealing that ANP32 is a deeply conserved pro-viral cofactor. They also show that sequence changes, start-site selection, and splice variants all contribute to diversifying proteins expressed from the ancestral *ANP32* locus. Finally, they highlight the remarkable plasticity of *Thogotoviruses*, which are able to utilize human, chicken, and tick ANP32 proteins that are separated by almost 1 billion years of evolution (36).

## MATERIALS AND METHODS

### Cell and plasmids

Authenticated 293T cells (CRL-3216) and Vero E6 cells (CRL-1586) were purchased from the American Type Culture Collection. 293T ANP32 knockout cells were generated by co-transfecting the Cas9-expressing plasmid pMJ920 (Addgene 42234) and two different sgRNA-expressing plasmids targeting ANP32A (target sites TCAGGTGAAAGAACTTGTCC and CTTTGGTAAGTTTGCGATTG) or ANP32B (target sites AGCCTACATTTATTAAACTG and ACAGGTTCGAGAACTTGTCT). Dual-knockout cells were created by co-transfecting all four different sgRNA-expressing plasmids. Cells were transfected with TransIT-X2 (Mirus MIR6005) in a 6-well plate using the recommended protocols. Twenty-four hours after transfection, clonal cell lines were generated by seeding 0.5 cells/well in a 96-well plate, expanding clones, and confirming the loss of ANP32A or ANP32B by blotting. All cell lines were grown at 37°C with 5% CO_2_ in Dulbecco’s modified Eagle’s medium supplemented with 10% heat-inactivated fetal bovine serum. Cells were regularly tested and verified free of *Mycoplasma* contamination using MycoAlert (Lonza LT07-318).

pCDNA3-based vector for A/WSN/1933 (H1N1) proteins was previously described (37–39). pCDNA3 expression vectors for NP, PB1, PB2, and PA were created by cloning genes from influenza B virus B/Brisbane/60/2008, influenza C virus C/Victoria/2/2012 (C/Vic), Bourbon virus strain STL, Thogoto virus strain SiAr126, and Dhori virus strain Indian/1313/61. Genes were cloned to encode C-terminal FLAG or V5 epitope tags. Viral polymerase reporter constructs were created by cloning firefly luciferase sequence, flanked by the 3′ and 5′ UTR sequence for each corresponding virus, into pHH21. DNA encoding cow ANP32A (NM_001195019.1), cow ANP32B (NP_001030246.1), chicken ANP32B (NP_001026105.1), brown dog tick ANP32A, and lone star tick ANP32A were synthesized (IDT) and cloned into pcDNA6.2 vector with a C-terminal FLAG tag. See below for the identification of tick ANP32 genes. Human ANP32A, human ANP32B, and chicken ANP32B were cloned from human and chicken cells into the pcDNA6.2 vector with a FLAG tag at the C-terminus (22).

### Identification of tick ANP32A

Syntenic *ANP32* gene clusters for humans, chickens, and swine were initially identified in the Kyoto Encyclopedia of Genes and Genomes Sequence Similarity DataBase (https://www.kegg.jp/kegg/genes.html). These were then used to search for *ANP32* genes and analogous clusters in the reference genome of *Rhipicephalus sanguineus* (BIME_Rsan_1.4). A similar approach was used for the *Amblyomma americanum* genome (ASM3014330v2), although this genome is not fully assembled or annotated yet, thus genes were identified manually in their corresponding contigs.

Tick ANP32A transcripts were identified by using Trinity (v2.15.1) to create *de novo* transcriptomes using RNA-seq data from *Rhipicephalus sanguineus* cell lines (BioProjects PRJNA238793-PRJNA238796) (40, 41). Transcripts were then mapped back to the locus to characterize splice variants. The same approach was used for *Amblyomma americanum* cell lines (BioProjects PRJNA238773, PRJNA238774, PRJNA238776, PRJNA238782, PRJNA238781, and PRJNA238784). Sequences for Rsa and Aam ANP32A variants can be found in Data S1.

### Viruses

Bourbon, Dhori, and Thogoto viruses were rescued from a 6-plasmid reverse genetic system based on that originally developed for influenza A virus (42). Gene segments were cloned from the genomes of Bourbon virus (strain BRBV-STL) (27), Thogoto virus strain SiAr126 (43), and Dhori virus strain Indian/1313/61 (44). Briefly, viral sequences corresponding to PB2, PB1, PA, GP, NP, and M were synthesized (Twist Bioscience) and cloned into the pWH2000 vector. The six genes for BRBV were PB2 (MK453529.1), PB1 (MK453528.1), PA (MK453527.1), GP (MK453526.1), NP (MK453525.1), and M (MK453524.1). For each strain, infectious virus was recovered by transfecting 1 µg of each plasmid into 293T using TransIT-LT1 (MIR2300, Mirus Bio). Cell culture supernatant was harvested 48 h later and amplified to create a working stock. Stocks from all three viruses were deep sequenced to confirm their identity and similarity to the deposited genomes.

### Growth curves and focus-forming assay

Wild-type 293T cells, huANP32A knockout 293T cells (AKO), huANP32B knockout 293T cells (BKO), and huANP32A and -B dual-knockout 293T cells (DKO), or DKO cells stably expressing huANP32A or -B were infected at a multiplicity of infection (MOI) of 0.01 for 1 h (BRBV) or an MOI of 1 for 2 h for DHOV with six replicates. Cells were then washed once with PBS and incubated at 37°C in Opti-MEM (Gibco) supplemented with 2% FBS (Sigma). Culture supernatants were collected at 24, 48, and 72 h post-infection, and viral titers were determined by focus-forming assays on Vero E6 cells. Infected cells were visualized using TMB substrate (Vector Laboratories) and quantified using an ImmunoSpot analyzer (Cellular Technologies).

### Polymerase activity assay

Wild type, AKO, BKO, and DKO 293T cells were transfected with plasmids encoding virus PA, PB1, PB2, and NP, a viral RNA-like firefly luciferase reporter, and a *Renilla* luciferase internal control reporter using TransIT-X2 (Mirus MIR6005). In this study, the polymerase activity assay was tested for the following viruses: influenza A virus (A/WSN/33), influenza B virus (B/Brisbane/60/2008), influenza C virus (C/Victoria/2/2012), Bourbon virus (strain BRBV-STL), Thogoto virus (strain SiAr126), and Dhori virus (strain Indian/1313/61). In some experiments, additional proteins like ANP32s were co-expressed as indicated. Firefly and *Renilla* luciferase activities were assayed ~24 h post-transfection. Firefly luciferase (FFLuc) activity was quantified with the Firefly Luciferase Assay (Promega E1500). Renilla luciferase (RLuc) activity was quantified with the Renilla-Glo Luciferase Assay (Promega E2720). FFLuc was normalized to RLuc within each sample. The expression levels of polymerase proteins in different cell lines were detected by western blotting.

### Western blotting and immunoprecipitation

Cells were lysed in co-immunoprecipitation (coIP) buffer (50 mM Tris pH 7.4, 150 mM NaCl, 0.5% NP40, protease inhibitor cocktail) and clarified by centrifugation. Co-immunoprecipitations were performed as before (22). Briefly, lysates were immunoprecipitated with anti-FLAG antibodies, and co-precipitating proteins were detected by blotting for the V5 epitope tag on the polymerase proteins. Samples were separated via denaturing polyacrylamide gel electrophoresis and transferred to polyvinylidene fluoride (PVDF) membranes prior to blocking and incubation with antibodies. Primary antibodies used were mouse α-tubulin DM1A (Proteintech 66031-1-Ig), α-actin (Proteintech 66009-1-Ig), α-human ANP32A (Proteintech 67687-1-Ig), α-human ANP32B (Proteintech 10843-1-AP), α-V5 (Chromotek v5ab), and M2 α-FLAG (Sigma F1804). Secondary antibodies were goat α-rabbit-HRP (Sigma A0545) or goat α-mouse-HRP (Sigma A4416), and chemiluminescent images were acquired with an Odyssey Fc Imager using Image Studio (LI-COR). Experiments were performed in triplicate, with representative images selected.

### Statistics

All experiments were repeated with at least three independent biological replicates, with at least three technical replicates within each experiment. Representative replicates are shown as the mean ± standard deviation. Pairwise comparisons were made using a two-tailed Student’s *t*-test. Multiple comparisons were performed with an analysis of variance (ANOVA) followed by an *ad hoc* Dunnett’s multiple comparisons test. Analyses were performed with Prism (v10.4.0, GraphPad).

## Data Availability

All associated data are present in the figures, and *de novo* transcripts are listed in Data S1.

## SUPPLEMENTAL MATERIAL

Supplemental material jvi.00522-25-s0001.pdf *De novo* transcripts.

ASM does not own the copyrights to Supplemental Material that may be linked to, or accessed through, an article. The authors have granted ASM a non-exclusive, world-wide license to publish the Supplemental Material files. Please contact the corresponding author directly for reuse.
